# The Importance of Social Support, Optimism and Resilience on the Quality of Life of Cancer Patients

**DOI:** 10.3389/fpsyg.2022.833176

**Published:** 2022-03-09

**Authors:** Iván Ruiz-Rodríguez, Isabel Hombrados-Mendieta, Anabel Melguizo-Garín, María José Martos-Méndez

**Affiliations:** Department of Social Psychology, Faculty of Psychology, University of Málaga, Málaga, Spain

**Keywords:** social support, quality of life, resilience, optimism, cancer, oncology

## Abstract

**Introduction:**

The aim of the present study is to carry out a multidimensional analysis on the relation between satisfaction with social support received, resilience and optimism in cancer patients and their quality of life.

**Materials and Methods:**

Data were gathered through questionnaires fulfilled by 142 cancer patients. Data relate to sociodemographic, health, quality of life, social support, resilience and optimism.

**Results:**

Satisfaction with the sources and types of support, resilience and optimism relates positively with quality of life. Predictive models show that informational support from friends is the variable that most increases patients’ general health, while emotional support from the partner is the one that best improves how patients cope with the disease. In the same line, emotional support from the partner, together with informational support from family are the ones that most contribute to reduce patients’ symptoms. Resilience improves general health and functioning, and reduces symptoms. Patients’ optimism and resilience also reduce symptoms. Gender differences were found, with females showing lower quality of life than males, mainly in how they cope with cancer. Patients in the stage of treatment showed lower quality of life and higher symptoms. Such increase was observed in patients who received hormonotherapy or chemotherapy.

**Discussion:**

Important practical implications can be drawn from results, which could help improve cancer patients’ quality of life through intervention strategies aimed at increasing their resilience, optimism and the social support provided by their closer sources.

## Introduction

In Spain, 300,000 new cases of cancer and more than 110,000 deaths were recorded in the year 2020 (World Health Organization. International Agency for Research on Cancer, 2021). Cancer is today among the main causes of death worldwide, accounting for almost 10 million deaths in 2020 (World Health Organization. International Agency for Research on Cancer, 2020).

Despite the latest therapeutic developments, the increase of early diagnosis and the number of cancer survivors, the overall medical process—including diagnosis and treatment—means patients face stressful events and situations (Bayly and Lloyd-Williams, 2016). Furthermore, cancer can become a chronic disease that forces both patients and their families to experience a prolonged wide range of stressful situations (Labrell et al., 2019). This is why cancer can deteriorate patients’ quality of life, affecting all aspects of their lives (Miller et al., 2016) and impacting their personal, social, and work life over extended periods of time.

### Quality of Life

Quality of life is a multidimensional construct defined as the extent to which patients’ expected physical, social, functional or emotional wellbeing is impacted by the medical process or treatment (Cella, 1994). It includes patients’ perceptions about the effects of the process of cancer, such as diagnosis and the different treatments administered (Polanski et al., 2016). Cancer reduces quality of life considerably (Pirri et al., 2013), due to being a disease in itself and the limitations experienced by patients (Mc Caughan et al., 2013). Such decrease in quality of life is caused by a variety of factors and it has been observed in every type of cancer (Barbus et al., 2018; Adamowicz et al., 2020) and all the stages of the medical process (Lopes et al., 2019).

Patients in the stage of follow-up fear a possible recurrence (Willems et al., 2016), which may lead to the reappearance of psychological, social and physical needs that have a negative impact on their quality of life. Many patients who overcame cancer find it difficult to go back to a new way of life, which can also impact their quality of life (Götze et al., 2015). Patients’ quality of life is affected by specific factors related to the medical process, such as the specific treatment administered (Marzorati et al., 2020), the location or type of cancer and the secondary effects derived from each treatment (Gargantini and Martín Casari, 2019; Nilsen et al., 2021), as well as sociodemographic factors such as patients’ gender. Males tend to perceive higher quality of life than females (Ayalon and Bachner, 2019). This difference may be caused by the different diagnoses in males and females (Franceschi and La Vecchia, 2001), or the treatments administered. For instance, females may experience physical changes that can make them feel less attractive and feminine (Abbott-Anderson and Kwekkeboom, 2012), thus reducing their quality of life (Lee et al., 2016). Assessing quality of life in cancer patients is key to successfully plan and control the treatment and therapeutic procedures, thus becoming a predictive factor for treatment thanks to its relation with higher quality of life and survival (Yan et al., 2016; Büttner et al., 2017).

### Social Support

Social support is key for patients to adapt to their new situation (Osann et al., 2014), and it relates with higher quality of life in cancer patients (Villanova Quiroga et al., 2018; Ruiz-Rodríguez et al., 2021a,b)—even in advanced cancer patients (Applebaum et al., 2014) and their relatives (Melguizo-Garín et al., 2019, 2020). In fact, the lack of social support relates to higher numbers of anxiety and depression cases (Fong et al., 2017), which leads to lower quality of life (Wells et al., 2014).

Social support is an interactive construct, an interpersonal transaction that takes place between a recipient of help and support sources. It implies emotions, information and material help in a specific context. Social support is often provided by the community, its social networks and its intimate relations both in everyday life situations and in times of crisis throughout individuals’ life (Lin et al., 1986). Most authors divide social support into three types (Breuer et al., 2017; Melguizo-Garín et al., 2019; Ruiz-Rodríguez et al., 2021a,b): emotional (feeling loved and having the certainty of having someone to trust), instrumental (availability of immediate help) and informational (receiving advice or information). There are instances where patients’ social support needs are not fulfilled due to the network not being prepared appropriately (Arora et al., 2007). Each type of support has a specific function (Lin, 1986), and satisfaction with support received is highly determined by patients’ specific needs.

Which source provides which type of support is key for patients to perceive such support as positive (Thoits, 1982). Patients tend to perceive friends and family—including their partners—as main sources of support (Doran et al., 2019; Ginter and Braun, 2019; Neris et al., 2020), and they consider support from these sources as essential to cope with the diagnosis and treatment (Pfaendler et al., 2015). This means that patients perceive support from these sources as essential for their life (Zhang et al., 2020). Some studies have analysed other sources of support, apart from the ones just mentioned. However, most of them do not include health professionals as sources of support, despite being a source in constant and close contact with patients throughout the disease and which can have a positive impact on patients’ quality of life (Ruiz-Rodríguez et al., 2021a).

Some studies confirm the positive relation between quality of life and social support (Kayser et al., 2018), despite not including multidimensional analyses of the sources and types of support received by patients. Analysing support from this perspective is relevant because patients’ needs for social support vary throughout the course of the disease and they allow to identify which types and sources of support relate to quality of life. If the specific type of support is not provided by the appropriate source needed by the patient, such support might be perceived as of little use (Breuer et al., 2017).

### Resilience

Results from research show a relation between cancer patients’ resilience and an increase in their quality of life (Oliva et al., 2019; Macía et al., 2020). Resilience is a dynamic construct, and it is defined as an individual’s ability to face adversity (Luthar et al., 2000), that is, stressful and traumatic events and overcome them in a positive and efficient manner. Resilience increases cancer patients’ health by helping them to cope better with negative emotions and feelings (Matzka et al., 2016), overall facing the disease better, the diagnosis and the treatment (Eicher et al., 2015). Highly resilient cancer patients show less negative symptoms, such as anxiety and depression (Smith et al., 2016). This evinces how these patients adapt better to the disease (Lam et al., 2017) and face it in a more adaptive way (Macía et al., 2020), which in turn leads to higher quality of life.

### Optimism

There are different approaches and perspectives in the study of optimism. Some studies understand optimism as individuals’ tendency to explain negative events and situations from the past in a temporarily limited way and as external cause to them. There is another approach, that of dispositional optimism, which would be more appropriate in the case of cancer due to the chronicity of the disease. Dispositional optimism has been defined as a relatively stable feeling of expecting general positive outcomes in life situations (Scheier and Carver, 1985). It is a generalised tendency to expect positive outcomes (Carver and Scheier, 2014) that impact individuals’ expectations in different areas of their lives. Dispositional optimism in cancer patients acts as a protective factor (Saboonchi et al., 2016) by boosting their adaption to the disease, and it relates to higher quality of life (Anderson et al., 2019; Marton et al., 2020). Low dispositional optimism relates to increased anxiety and depression, thus leading to lower quality of life (Schou et al., 2004). Conversely, optimist cancer patients experience a decrease in their anxiety and depression over time and their wellbeing and quality of life increases gradually (Fischer et al., 2018; Jimenez-Fonseca et al., 2018).

### Present Study

The aim of the present study is to analyse cancer patients’ satisfaction with social support, their quality of life, dispositional optimism and resilience. More specifically, the aim is to analyse whether patients’ satisfaction with social support, dispositional optimism and resilience relates positively to their quality of life considering the treatment received, the different stages of the disease and gender differences. The present study contributes with a multidimensional analysis of cancer patients’ satisfaction with social support provided by four different sources of support (friends, partner, family and health professionals), as well as the three types of support provided previously mentioned (emotional, instrumental and informational). This multidimensional approach provides greater and deeper knowledge on the variables that affect quality of life, thus enabling the design and development of more specific intervention strategies.

The hypotheses suggested are the following: (1) patients undergoing treatment perceive lower quality of life than patients in the stage of follow-up; (2) satisfaction with emotional, instrumental and informational support provided by family, partner, friends and health professionals, optimism and resilience relate positively to patients’ quality of life.

## Materials and Methods

### Participants and Procedure

The study participants were 142 cancer patients, aged between 22 and 48, with an age mean of 48.96 (DT = 13.60). Patients were at different stages of the medical process and suffered from different types of cancer (Table 1). 23.2% of patients were males (33) and 76.8% were females (109). Patients were members of different associations in Spain and received treatment at the Costa del Sol Hospital (Marbella). Inclusion criteria were the following: cancer patients over 18 undergoing treatment or in follow-up stage. Conversely, exclusion criteria were underage cancer patients who had completed the stages of follow-up or revision.

**Table 1:** Sociodemographic and health variables (*n* = 142).

Age	48.96 (13.60)^a^
Gender	**% (N)**
Male	23.2 (33)
Female	76.8 (109)
Marital status	
Single	19.0 (27)
Married	62.0 (88)
Divorced/separated	7.0 (10)
Widow	3.5 (5)
Domestic partner	8.5 (12)
Education level	
University or higher completed	37.3 (53)
Undergoing university studies or higher	3.5 (5)
A levels or vocational training completed	26.8 (38)
Compulsory secondary education completed	28.2 (40)
None of the above	4.2 (6)
Treatment stage	
Undergoing treatment	50.0 (71)
Follow-up	50.0 (71)
Cancer type	
Thyroid	45.7 (63)
Breast	22.5 (32)
Colon	9.4 (13)
Lung	4.3 (6)
Ovary	2.9 (4)
Other	15.2 (20)
Chemotherapy treatment	
No	49.6 (70)
Yes	50.4 (71)
Radiotherapy treatment	
No	44.7 (63)
Yes	55.3 (78)
Surgical treatment	
No	17.7 (25)
Yes	82.3 (116)
Hormonotherapy treatment	
No	77.3 (109)
Yes	22.7 (32)
Time since initial diagnosis	
Less than 1 year	33.1 (46)
1–2 years	24.5 (34)
2–5 years	26.6 (37)
Over 5 years	15.8 (22)

a *Mean (Standard deviation)*.

The study was approved by the Ethical Committee of the hospital since interviews were carried out with patients at the hospital facilities after patients’ appointments with their doctors. Patients were informed about the purpose of the study before each interview, and they were guaranteed anonymity and confidentiality through an informed consent form. A website was also created for patients who lived outside of Málaga so they could also participate (this website also contained information about the purpose of the study and participants’ anonymity and confidentiality).

The Ethical Committee of the University of Málaga, along with the Ethical Committee of the Costa del Sol Hospital, agreed to the study’s accordance with legal and ethical methodological criteria for its execution (reference number: CEUMA-58-2016-H).

### Instruments

#### Sociodemographic and Health Questionnaire

Health (stage of treatment, type of cancer, hormonotherapy treatment, chemotherapy treatment, radiotherapy treatment, surgical treatment and time since initial diagnosis) and sociodemographic data (age, gender, marital status and educational level) were collected.

#### Quality of Life Questionnaire (EORTC-QLQ C30-European Organization for Research and Treatment of Cancer)

This questionnaire is an adapted version for Spanish-speaking population (Arrarás et al., 1995) and it assesses different areas of quality of life reported by cancer patients (Aaronson et al., 1993). It comprises 28 Likert-type items ranging from 1 to 4 (1 being ‘None at all’ and 4 ‘Very much’), and 2 Likert-type items ranging from 1 to 7 (1 being ‘Terrible’ and 7 ‘Excellent’).

This questionnaire provides a global health scale (overall assessment of health and quality of life), five functioning scales (physical, role-wise, emotional, cognitive and social), three symptom scales (fatigue, nausea/vomiting and pain) and six items (loss of appetite, dyspnoea, constipation, diarrhoea, insomnia and financial difficulties).

Scores from each scale are grouped in a total final score from 0 to 100. Higher scores in the functioning scales and the global health scale indicate higher quality of life. Higher scores in the symptom scales indicate lower quality of life. The final scale shows Cronbach Alpha reliability of *α* = 0.86. The global health scale shows Cronbach Alpha reliability of *α* = 0.90. Functioning scales show the following Cronbach Alpha: physical (0.77), role-wise (0.85), emotional (0.85), cognitive (0.71) and social (0.86). Symptom scales show the following Cronbach Alpha: fatigue (0.88), nausea/vomiting (0.73) and pain (0.86).

#### Questionnaire of Frequency and Satisfaction With Social Support

This questionnaire comprises 12 items that measure satisfaction with social support from a multidimensional approach differentiating between the three types of support (emotional, instrumental and informational) provided by the four sources (García-Martín et al., 2016). The study includes family, partner, friends and health professionals as sources of support to assess patients’ satisfaction with the different types of support, as indicated by the authors of the instrument and according to the context in which it is used. This questionnaire has showed high levels of reliability when used with different sources of support in different populations, such as relatives of cancer patients (Melguizo-Garín et al., 2019).

It uses a Likert-type scale from 1 to 5 (1 being ‘Dissatisfied’ and 5 ‘Very satisfied’) to assess and measure cancer patients’ satisfaction with emotional, instrumental and informational support received from their partner, family, friends and health professionals. This scale shows Cronbach Alpha reliability of *α* = 0.93.

#### 10-Item Connor-Davidson Resilience Scale (CD-RISC 10)

This is a shortened version of the original scale (Connor and Davidson, 2003). It comprises 10 Likert-type items ranging from 0 to 4 (0 being ‘Never’ and 4 ‘Almost always’) (Davidson, 2018). The final score is the sum of the scores obtained from each item. The final score ranges from 0 to 40. High scores indicate higher resilience. This scale shows Cronbach Alpha reliability of *α* = 0.90.

#### Life Orientation Test Revised

This questionnaire has been adapted to Spanish-speaking population (Otero et al., 1998) and it comprises six items (along with four distractors) in a Likert-type scale of 5 points (Scheier et al., 1994). Three items are written positively (pole of optimism) and three items are written negatively (pole of pessimism). The score obtained from the three negative items is reverted to achieve a final score oriented towards optimism. Therefore, high scores indicate higher dispositional optimism. Internal consistency of the instrument is *α* = 0.78.

### Statistical Analysis

Data were analysed through IBM SPSS version 23, using descriptive statistics to analyse sociodemographic and health variables.

In order to test the study’s hypotheses, the following analyses were carried out as: Pearson’s correlation coefficient, Student’s tests and multiple linear regression analysis.

Pearson’s correlation coefficient was applied to determine the relation between quantitative variables such as satisfaction with social support, differentiating the types of support (emotional, instrumental and informational) and the sources of support (family, partner, friends and health professionals), resilience and optimism from the dimensions of quality of life (overall health status, coping scales and symptom scales/items).

Student’s *t*-tests were carried out on gender variables (male/female), chemotherapy (yes/no), radiotherapy (yes/no), surgical treatment (yes/no), hormonotherapy (yes/no) and stage of treatment (undergoing treatment/follow-up) to compare means from the previous quantitative variables to these variables.

Variables that showed significant results in the Pearson’s analysis and the Student’s t-tests were included as independent variables in the multiple linear regression analysis. Each scale/item of quality of life was used as dependent variable in the models.

## Results

Data from the overall sample in the areas of quality of life, satisfaction with social support received, dispositional optimism and resilience are shown in Table 2. Scores from scales/items of quality of life are satisfactory according to their high scores in the global health scale and the functioning scales, and low in the scales/items related to symptoms. Satisfaction with emotional, instrumental and informational social support from family, partner, friends and health professionals, as well as optimism and resilience are also high.

**Table 2:** Mean and standard deviations from questionnaires.

EORTC QLQ-C30	Mean (Standard Deviation)
Global health scale	63.73 (21.88)
**Functional scales**	
Physical functioning	81.88 (18.23)
Role functioning	75.70 (28.46)
Emotional functioning	68.78 (24.50)
Cognitive functioning	76.06 (25.30)
Social functioning	71.48 (29.28)
**Symptom scales**	
Fatigue	38.73 (26.82)
Nausea and vomiting	6.57 (13.31)
Pain	26.06 (26.22)
Dyspnoea	12.44 (22.67)
Insomnia	35.45 (32.55)
Loss of appetite	11.50 (23.83)
Constipation	15.73 (25.01)
Diarrhoea	11.50 (21.76)
Financial difficulties	23.00 (31.31)
**QFSSS**	
Satisfaction with emotional support from family	3.99 (1.23)
Satisfaction with instrumental support from family	4.02 (1.23)
Satisfaction with informational support from family	3.64 (1.33)
Satisfaction with emotional support from partner	3.92 (1.32)
Satisfaction with instrumental support from partner	4.04 (1.25)
Satisfaction with informational support from partner	3.73 (1.38)
Satisfaction with emotional support from friends	3.79 (1.34)
Satisfaction with instrumental support from friends	3.54 (1.43)
Satisfaction with informational support from friends	3.44 (1.44)
Satisfaction with emotional support from health professionals	3.65 (1.49)
Satisfaction with instrumental support from health professionals	3.68 (1.42)
Satisfaction with informational support from health professionals	3.73 (1.45)
**LOT-R**	63.23 (18.17)
**CD-RISC 10**	30.49 (7.59)

### Mean Differences

Tables 3 and 4 show the differences between means in the global health scale, the functioning scales and the scales/items related to symptoms based on gender, type of treatment received and stage of the medical process. Results show statistically significant differences. Females show lower physical and cognitive functioning, as well as higher fatigue. Regarding the first hypothesis suggested, patients who are undergoing treatment also show lower physical, role and cognitive functioning, as well as higher loss of appetite and financial difficulties. Patients who received surgical treatment show lower cognitive functioning and higher gastrointestinal problems. Those patients who received radiotherapy show higher emotional and cognitive functioning, but also higher fatigue, dyspnoea and constipation. Those patients who received chemotherapy show higher levels of insomnia than those patients who did not. Finally, patients who received hormonotherapy perceive overall lower health and physical functioning, higher fatigue, pain and financial difficulties.

**Table 3:** Mean differences in the global health scale, functioning scales based on gender, stage of medical process and type of treatment.

	Global health		Physical functioning		Role functioning		Emotional functioning		Cognitive functioning		Social functioning	
	M (DT)	*t*	M (DT)	*t*	M (DT)	*t*	M (DT)	*t*	M (DT)	*t*	M (DT)	*t*
**Gender**												
Male	69.19 (21.90)	0.102	87.07 (13.53)	0.027^*^	81.82 (26.47)	0.160	74.24 (25.97)	0.144	88.89 (17.51)	0.000^*^	75.76 (30.64)	0.340
Female	62.08 (21.71)		80.31 (19.21)		73.85 (28.90)		67.13 (23.92)		72.17 (26.06)		70.18 (28.87)	
**Stage**												
Follow-up	66.20 (20.16)	0.180	86.48 (15.03)	0.002^*^	80.99 (24.93)	0.026^*^	67.14 (25.43)	0.426	71.83 (26.37)	0.046^*^	74.41 (27.58)	0.234
Undergoing treatment	61.27 (23.36)		77.28 (20.02)		70.42 (30.89)		70.42 (23.60)		80.28 (23.62)		68.54 (30.80)	
**Surgical treatment**												
No	60.00 (21.11)	0.338	81.07 (21.23)	0.837	72.67 (34.32)	0.581	77.33 (17.76)	0.052	90.00 (17.35)	0.000^*^	80.00 (25.00)	0.101
Yes	64.66 (22.11)		81.90 (17.62)		76.15 (27.21)		66.81 (25.47)		72.84 (25.78)		69.40 (29.89)	
**Radiotherapy**												
No	64.55 (21.74)	0.727	81.80 (18.25)	0.977	73.28 (39.90)	0.401	74.07 (21.64)	0.018^*^	81.48 (22.22)	0.018^*^	71.16 (28.90)	0.967
Yes	63.25 (22.21)		81.71 (18.34)		77.35 (26.45)		64.32 (26.00)		71.37 (26.85)		71.37 (29.78)	
**Chemotherapy**												
No	64.05 (21.21)	0.907	82.29 (17.98)	0.730	79.76 (24.56)	0.080	66.43 (24.57)	0.282	73.10 (25.11)	0.195	72.14 (28.76)	0.729
Yes	63.62 (22.77)		81.22 (18.60)		71.36 (31.52)		70.89 (24.51)		78.64 (25.38)		70.42 (29.97)	
**Hormonotherapy**												
No	66.28 (20.89)	0.014^*^	83.67 (17.88)	0.020^*^	77.52 (29.43)	0.126	70.80 (24.52)	0.058	78.13 (24.92)	0.051	72.63 (29.97)	0.313
Yes	55.47 (23.63)		75.21 (18.18)		68.75 (24.23)		61.46 (23.64)		68.23 (25.53)		66.67 (26.77)	

* *p ≤ 0.05*.

**Table 4:** Mean differences in symptoms scales/items based on gender, stage of medical process and type of treatment.

	Fatigue		Nausea and vomiting		Pain		Dyspnoea		Insomnia		Loss of appetite		Constipation		Diarrhoea		Financial difficulties	
	M (DT)	*t*	M (DT)	*t*	M (DT)	*t*	M (DT)	*t*	M (DT)	*t*	M (DT)	*t*	M (DT)	*t*	M (DT)	*t*	M (DT)	*t*
**Gender**																		
Male	27.61 (22.42)	0.006^*^	6.06 (10.05)	0.802	20.71 (26.03)	0.182	9.09 (17.23)	0.334	31.31 (35.30)	0.407	16.16 (27.79)	0.201	16.16 (31.32)	0.910	13.13 (24.92)	0.625	25.25 (36.35)	0.639
Female	42.10 (27.22)		6.73 (14.19)		27.68 (26.18)		13.46 (24.05)		36.70 (31.73)		10.09 (22.45)		15.60 (22.94)		11.01 (20.81)		22.32 (29.76)	
**Stage**																		
Follow-up	38.50 (26.81)	0.917	4.93 (13.63)	0.142	24.18 (25.94)	0.395	9.86 (19.02)	0.176	34.74 (32.09)	0.798	5.63 (12.58)	0.003^*^	12.68 (21.36)	0.147	7.98 (18.23)	0.054	17.37 (28.09)	0.031^*^
Undergoing treatment	38.97 (27.01)		8.22 (12.87)		27.93 (26.54)		15.02 (25.69)		36.15 (33.21)		17.37 (30.27)		18.78 (28.02)		15.02 (24.42)		28.64 (33.47)	
**Surgical treatment**																		
No	38.67 (31.77)	0.983	8.00 (10.89)	0.570	20.67 (27.76)	0.243	9.33 (24.57)	0.440	37.33 (29.38)	0.753	17.33 (30.61)	0.288	16.00 (27.42)	0.972	5.33 (12.47)	0.024^*^	18.67 (29.00)	0.479
Yes	38.79 (25.90)		6.32 (13.84)		27.44 (25.84)		13.22 (22.36)		35.06 (33.43)		10.34 (22.16)		15.80 (24.66)		12.93 (23.27)		23.56 (31.70)	
**Radiotherapy**																		
No	33.16 (24.88)	0.025^*^	6.35 (12.13)	0.830	21.96 (24.10)	0.081	8.47 (17.93)	0.048^*^	32.28 (29.92)	0.300	12.70 (27.06)	0.620	20.63 (30.19)	0.051^*^	9.52 (19.33)	0.315	23.28 (33.14)	0.842
Yes	43.30 (27.78)		6.84 (14.32)		29.70 (27.48)		15.81 (25.61)		38.03 (34.70)		10.68 (21.15)		11.97 (19.35)		13.25 (23.63)		22.22 (29.75)	
**Chemotherapy**																		
No	39.52 (26.12)	0.743	5.71 (11.66)	0.426	26.67 (27.28)	0.849	11.90 (23.42)	0.747	30.00 (27.31)	0.048^*^	10.00 (21.50)	0.437	12.86 (19.07)	0.161	13.81 (23.05)	0.230	18.10 (28.20)	0.082
Yes	38.03 (27.84)		7.51 (14.85)		25.82 (25.32)		13.15 (22.17)		40.85 (36.60)		13.15 (26.11)		18.78 (29.67)		9.39 (20.46)		27.23 (33.48)	
**Hormonotherapy**																		
No	35.98 (25.74)	0.023^*^	6.88 (13.84)	0.669	21.56 (25.18)	0.000^*^	11.01 (21.78)	0.143	32.72 (21.42)	0.066	11.32 (24.10)	0.806	14.68 (25.02)	0.312	10.70 (21.22)	0.378	18.65 (29.20)	0.004^*^
Yes	48.26 (20.00)		5.73 (11.68)		42.19 (23.56)		17.71 (25.38)		44.79 (35.53)		12.50 (23.57)		19.79 (25.20)		14.58 (23.85)		36.46 (34.24)	

* *p ≤ 0.05*.

### Relation Between Variables

Correlation coefficients are shown in Tables 5 and 6. Based on satisfaction with social support received, global health related positively with all the types of support received from all sources, except instrumental support received from family. Regarding functioning scales, emotional and cognitive functioning related positively with all types and sources of support received; physical support related positively with emotional support received from the partner; and finally, social functioning related positively with satisfaction with emotional support received from family and partner. All types and sources of support in the symptom scales/items related to less fatigue; all types of support provided by the partner related to less dyspnoea; it was observed that emotional support from the partner decreased nausea, vomiting and gastrointestinal issues; emotional support received from family and instrumental and informational support from health professionals relates to less pain; informational support from family relates to less insomnia; emotional and instrumental support from the partner relates to less gastrointestinal issues; and, finally, informational support from friends relates to less financial difficulties.

**Table 5:** Relation between satisfaction with social support and quality of life.

	Satisfaction with social support received											
	Family			Partner			Friends			Health professionals		
EORTC QLQ-C30	Emo	Instru	Info	Emo	Instru	Info	Emo	Instru	Info	Emo	Instru	Info
Global health	0.247^**^	138	0.176^*^	0.248^**^	0.154^*^	0.191^*^	0.255^**^	0.225^**^	0.258^**^	0.150^*^	0.176^*^	−0.277^**^
**Functioning scales**												
Physical functioning	0.128	0.036	0.082	0.207^*^	0.083	0.151	0.065	0.022	0.053	0.031	0.095	0.063
Role functioning	−0.017	−0.130	−0.070	0.076	−0.022	−0.034	0.032	0.007	0.002	0.030	0.022	0.006
Emotional functioning	0.249^**^	0.210^**^	0.240^**^	0.305^**^	0.231^**^	0.190^*^	0.170^*^	0.216^**^	0.207^**^	0.268^**^	0.231^**^	0.241^**^
Cognitive functioning	0.290^**^	0.266^**^	0.279^**^	0.321^**^	0.220^**^	0.266^**^	0.212^**^	0.214^**^	0.184^*^	0.319^**^	0.334^**^	0.313^**^
Social functioning	0.147^*^	0.004	0.039	0.200^*^	0.132	0.075	0.110	0.067	0.084	0.120	0.091	0.104
**Symptom scale**												
Fatigue	−0.189^*^	−0.139^*^	−0.190^*^	−0.340^**^	−0.239^**^	−0.233^**^	−0.148^*^	−0.153^*^	−0.165^*^	−0.184^*^	−0.226^**^	−0.205^**^
Nausea and vomiting	0.006	−0.001	0.034	−0.162^*^	−0.115	−0.105	−0.014	−0.002	0.032	0.032	0.011	0.021
Pain	−0.154^*^	−0.127	−0.089	−0.108	−0.061	0.040	−0.094	−0.083	−0.052	−0.138	−0.139^*^	−0.165^*^
Dyspnoea	−0.053	−0.018	−0.117	−0.353^**^	−0.219^**^	−0.239^**^	−0.030	−0.020	0.004	−0.005	−0.024	−0.039
Insomnia	−0.130	−0.113	−0.162^*^	−0.130	−0.088	−0.107	−0.066	−0.096	−0.066	−0.073	−0.042	−0.073
Loss of appetite	−0.019	0.064	−0.070	−0.088	0.012	−0.022	−0.012	0.003	−0.040	−0.014	−0.010	−0.031
Constipation	−0.062	−0.003	0.050	−0.154^*^	−0.047	−0.012	−0.048	−0.035	0.008	0.045	−0.032	0.042
Diarrhoea	0.015	0.079	−0.036	−0.238^**^	−0.181^*^	−0.116	−0.054	−0.042	_-0.066	−0.052	−0.019	−0.109
Financial difficulties	−0.022	0.055	−0.010	−0.067	−0.054	−0.026	−0.126	−0.101	−0.160^*^	−0.026	−0.021	−0.006

* *p ≤ 0.05*;

** *p* ≤ 0.01.

**Table 6:** Relation between age, time since diagnosis, resilience and optimism variables and quality of life.

EORTC QLQ-C30	Age	Time since initial diagnosis	Resilience	Optimism
Global health	0.121	−0.022	0.377^**^	0.271^**^
**Functioning scales**				
Physical Functioning	0.046	−0.025	0.143^*^	0.130
Role functioning	0.075	0.022	0.195^*^	0.171^*^
Emotional functioning	0.173^*^	0.017	0.497^**^	0.408^**^
Cognitive functioning	0.231^**^	−0.099	0.383^**^	0.265^**^
Social functioning	0.107	−0.004	0.480^**^	0.271^**^
**Symptom scale**				
Fatigue	−0.152^*^	0.048	−0.325	−0.277^**^
Nausea and vomiting	0.034	−0.041	−0.069	−0.106
Pain	−0.002	0.201^**^	−0.170^*^	−0.162^*^
Dyspnoea	−0.082	−0.058	−0.181^*^	−0.211^**^
Insomnia	−0.045	0.063	−0.201^**^	−0.159^*^
Loss of appetite	0.158^*^	−0.061	−0.317^**^	−0.092
Constipation	0.084	0.006	−0.073	0.029
Diarrhoea	−0.171^*^	−0.008	−0.253^**^	−0.181^*^
Financial diff.	−0.083	−0.069	−0.195^*^	−0.103

* *p ≤ 0.05*;

** *p* ≤ 0.01.

The higher patients’ age the higher their emotional and cognitive functioning, as well as less fatigue and gastrointestinal issues and lower loss of appetite. Results show that the longer the time from the initial diagnosis, the lower the pain. Higher resilience in patients related to overall better health, better functioning in all areas and less symptoms such as pain, dyspnoea, loss of appetite, gastrointestinal problems and financial difficulties. Higher optimism also related to better health, better role, emotional, cognitive and social functioning, and less symptoms such as fatigue, pain, dyspnoea, loss of appetite and gastrointestinal problems.

### Predictive Models for Quality of Life

Regarding the second hypothesis of the study, regression analyses were carried out to identify potential predictive factors of the different dimensions of quality of life. The global health scale, functioning scales and symptom scales/items were used as dependent variables in each model. In the equations, sociodemographic and health variables that were significant in the previous analyses carried out were used as predictors, as well as the variables of resilience, optimism and satisfaction with emotional, instrumental and informational support from family, friends, partner and health professionals that showed significant values in the correlation analyses previously performed. Tables 7 and 8 show the final regression models for each dependent variable.

**Table 7:** Multiple lineal regression analysis for global health and functioning scales (*n* = 142).

Model	Non-standardised coefficients		Standardised coefficients	*t*	Sig.
	B	Standard error	Beta		
**Global health**
(constant)	26.268	7.341		3.578	<0.001^**^
Resilience	0.952	0.234	0.330	4.074	<0.001^**^
Informational friends	2.455	1.234	0.161	1.990	0.049^*^
*R* = 0.408 *R*^2^ = 0.166 *R*^2^ adjusted = 0.154 *F* = 13.841 Sig. = <0.001^**^					
**Physical functioning**					
(constant)	96.844	9.754		9.929	<0.001^**^
Emotional partner	2.888	1.216	0.205	2.375	0.019^*^
Gender	−11.165	4.000	−0.257	−2.792	0.006^**^
Stage of treatment procedure	−13.828	3.370	−0.374	−4.103	<0.001^**^
*R* = 0.424 *R*^2^ = 179 *R*^2^ adjusted =0.158 *F* = 8.310 Sig. = <0.001^**^					
**Role functioning**					
(constant)	56.380	9.630		5.855	<0.001^**^
Resilience	0.834	0.307	0.222	2.715	0.007^**^
Stage of medical process	−12.196	4.646	−0.215	−2.625	0.010^*^
*R* = 0.289 *R*^2^ = 0.083 *R*^2^ adjusted = 0.070 *F* = 6.315 Sig. = 0.002^**^					
**Cognitive functioning**					
(constant)	69.694	8.303		8.394	<0.001^**^
Emotional partner	5.466	1.527	0.306	3.580	0.001^**^
Surgical treatment	−16.288	5.353	−0.260	−3.043	0.003^**^
*R* = 0.433 *R*^2^ = 0.187 *R*^2^ adjusted = 0.173 *F* = 13.130 Sig. = <0.001^**^					

* *p* ≤ 0.05

** *p* ≤ 0.01;

*Gender: 0 = male and 1 = female; stage of medical process: 0 = Follow-up and 1 = Undergoing treatment; and Surgical treatment: 0 = No and 1 = Yes*.

**Table 8:** Multiple lineal regression analysis for symptom scales/items (*n* = 142).

Model	Non-standardised coefficients		Standardised coefficients	*t*	Sig.
	*B*	Standard error	Beta		
**Fatigue**
(constant)	85.799	10.389		8.258	<0.001^**^
Resilience	−0.849	0.319	−0.238	−2.666	0.009^**^
Emotional partner	−5.381	1.794	−0.268	−3.000	0.003^**^
*R* = 0.409 *R*^2^ = 0.167 *R*^2^ adjusted = 0.153 *F* = 11.547 Sig. = <0.001^**^					
**Pain**					
(constant)	21.560	2.379		9.064	<0.001^**^
Hormonotherapy	20.628	4.993	0.331	4.132	<0.001^**^
*R* = 0.331 *R*^2^ = 0.109 *R*^2^ adjusted = 0.103 *F* = 17.070 Sig. = <0.001^**^					
**Dyspnoea**					
(constant)	29.111	6.780		4.294	<0.001^**^
Optimism	−0.264	0.103	−0.211	−2.557	0.012^**^
*R* = 0.211 *R*^2^ = 0.045 *R*^2^ adjusted = 0.038 *F* = 6.540 Sig. = 0.012^*^					
**Insomnia**					
(constant)	72.230	11.867		6.086	<0.001^**^
Resilience	−0.881	0.355	−0.205	−2.481	0.014^*^
Informational family	−5.280	2.129	−0.216	−2.480	0.014^*^
Chemotherapy treatment	18.321	5.608	0.281	3.267	0.001^**^
*R* = 0.351 *R*^2^ = 0.123 *R*^2^ adjusted = 0.104 *F* = 6.414 Sig. = <0.001^**^					
**Loss of appetite**					
(constant)	38.547	7.603		5.070	<0.001^**^
Resilience	−1.115	0.242	−0.355	−4.600	<0.001^**^
Stage of treatment procedure	13.921	3.668	0.293	3.795	<0.001^**^
*R* = 0.430 *R*^2^ = 0.185 *R*^2^ adjusted = 0.173 *F* = 15.788 Sig. = <0.001^**^					
**Diarrhoea**					
(constant)	33.620	7.363		4.566	<0.001^**^
Resilience	−0.726	0.234	−0.253	−3.095	0.002^**^
*R* = 0.253 *R*^2^ = 0.074 *R*^2^ adjusted = 0.057 *F* = 9.579 Sig. = 0.002^**^					
**Economic difficulties**					
(constant)	35.829	10.737		3.337	0.001^**^
Resilience	−0.796	0.332	−0.194	−2.394	0.018^*^
Stage of treatment procedure	14.577	4.983	0.234	2.925	0.004^**^
Hormonotherapy	16.612	5.981	0.224	2.777	0.006^**^
*R* = 0.371 *R*^2^ = 0.138 *R*^2^ adjusted = 0.119 *F* = 7.300 Sig. = <0.001^**^					

* *p* ≤ 0.05;

** *p* ≤ 0.01;

*Gender: 0 = male and 1 = female; Stage of medical process: 0 = Follow-up and 1 = Undergoing treatment; Chemotherapy Treatment: 0 = No and 1 = Yes; and Hormonotherapy: 0 = No and 1 = Yes*.

Through the regression analysis, significant models were revealed for one part of the variance in different dimensions of quality of life (global health, symptom scales/items and functioning scales). The regression equation for global health shows *R*^2^ = 0.166, *F* = 13,841, *p* < 0.001. Resilience (*β* =0.330, *p* <0.001) and satisfaction with informational support from friends (*β* = 0.161, *p* =0.049) relate to an increase in cancer patients’ global health. Regarding functioning scales, the regression equation of physical functioning showed *R*^2^ =0.179, *F* = 8,310, *p* <0.001. Male gender (*β* = −0.257, *p* = 0.006), the stage of follow-up (*β* = −0.374, *p* = <0.001) and satisfaction with emotional support from the partner (*β* = 0.205, *p* = 0.019) predict higher physical functioning. The regression equation for role functioning showed *R*^2^ =0.083, *F* = 6,315, *p* = 0.002. Resilience (*β* = 0.222, *p* = 0.007) and being in the stage of follow-up (*β* = −0.215, *p* =0.010) contribute to a better role functioning. The regression equation for cognitive functioning showed *R*^2^ = 0.187, *F* = 13,130, *p* <0.001. Satisfaction with emotional support from the partner increases cognitive functioning (*β* = 0.306, *p* = 0.001). Conversely, having received surgical treatment (*β* = −0.260, *p* = 0.003) predicts lower cognitive functioning. Finally, results did not show significant predictive models for patients’ emotional and social functioning.

Regarding the symptom scale, fatigue showed *R*^2^ = 0.167, *F* = 11.547, *p* < 0.001. Resilience (*β* = −0.238, *p* = 0.009) and satisfaction with emotional support from partner (*β* = −0.268, *p* = 0.003) reduce fatigue in patients. The regression equation for pain showed *R*^2^ = 0.109, *F* = 17.070, *p* < 0.001. Receiving hormonotherapy (*β* = 0.331, *p* <0.001) predicts an increase in pain. The regression equation for dyspnoea showed *R*^2^ = 0.045, *F* = 6.540, *p* = 0.012. Optimism (*β* = −0.211, *p* = 0.012) predicts a decrease in dyspnoea. The regression equation for insomnia showed *R*^2^ = 0.123, *F* = 6.414, *p* < 0.001. Patients’ resilience (*β* = −0.205, *p* = 0.014), satisfaction with support (*β* = −0.216, *p* = 0.014) and not having received chemotherapy (*β* = 0.281, *p* = 0.001) reduce insomnia. The regression equation for loss of appetite showed *R*^2^ = 0.185, *F* = 15.788, *p* < 0.001. Resilience (*β* = −0.355, *p* < 0.001) and being in the follow-up stage (*β* = 0.293, *p* < 0.001) predict lower loss of appetite. The regression equation for diarrhoea showed *R*^2^ = 0.074, *F* = 9.579, *p* = 0.002. Resilience (*β* = −0.253, *p* = 0.002) predicts less diarrhoea in patients. The regression equation for economic difficulties showed *R*^2^ = 0.138, *F* = 7.300, *p* < 0.001. Resilience (*β* = −0.194, *p* = 0.018) predicts lower economic difficulties. However, being undergoing treatment (*β* = 0.234, *p* = 0.004) and having received hormonotherapy (*β* = 0.224, *p* = 0.006) predict higher economic difficulties. Finally, no significant models were found to predict nausea/vomiting and constipation.

## Discussion

The present study proposes an analysis of the relation between the different sources of support and types of social support, dispositional optimism and resilience and cancer patients’ quality of life. It considers gender differences, type of treatment received and the stage of the medical process. Overall, results confirm the hypotheses initially suggested.

The first hypothesis suggested was that patients who are undergoing treatment perceive lower quality of life than those who are in follow-up. The study’s results show that, in general, patients who are undergoing treatment recorded lower scores in different dimensions of quality of life. The toxicity of treatments can increase symptoms and the subsequent secondary effects, which in turn relates to a decrease in patients’ quality of life (Zhang et al., 2020). Also, many patients who are undergoing treatment have previously received surgical interventions. This means that they might have to adapt to a permanent disability potentially caused by such interventions, thus making it a potential traumatic experience with a high impact on their quality of life (Mahjoubi et al., 2010), as opposed to those patients in follow-up, who have already been through such experiences. Results show that patients who are undergoing treatment experience higher economic difficulties than those in follow-up. Along with the increased expenses due to the disease itself, patients sometimes must face losing their employment, thus implying an even higher loss of income (Pearce et al., 2015), which is known to be related to a decrease in quality of life (Lu et al., 2019). There is one striking result related to patients’ functioning: those undergoing treatment show better functioning than follow-up patients. This might be caused by the fact that patients in follow-up might be experiencing a decrease in the support provided by their networks due to the main treatment being completed, which can in turn impact their quality of life. Furthermore, patients in follow-up fear a potential recurrence of the disease (Willems et al., 2016), which might contribute to a decrease in their cognitive functioning.

The second hypothesis suggested is related to cancer patients’ satisfaction with social support, dispositional optimism and resilience and their positive impact on quality of life. Results obtained confirm such hypothesis. A lack in social support lowers patients’ quality of life to such an extent that the loss or absence of support networks from family or friends can lead to an increase of the mortality risk after the cancer diagnosis (Lin, 2016). The lack of support after the diagnosis and the subsequent treatment can have a negative impact on the effect of the treatment itself (Thompson et al., 2017). In fact, research notes the importance of social support to increase positive outcomes of cancer treatments (Spatuzzi et al., 2016; Thompson et al., 2017). Social support contributes to higher psychological adjustment (Yağmur and Duman, 2016) and lower risk of depression (Fong et al., 2017; Hsieh et al., 2020). Social support acts as a protective factor for quality of life, being an important predictor of quality of life in cancer patients (Yoon et al., 2018; Shen et al., 2020; Zhang et al., 2020), both for those undergoing treatment (Applebaum et al., 2014) and those who have already overcome it (Leung et al., 2014). Results are in line with studies that suggest higher resilience in patients relates to higher quality of life (Oliva et al., 2019) and lower risk of depression (Sharpley et al., 2014). Resilience helps patients cope better with negative emotions and feelings (Matzka et al., 2016), as well as to face the disease in an adaptive way, which in turn increases their quality of life (Macía et al., 2020). Some authors have noted the importance of resilience in cancer patients as it helps them have more active coping mechanisms before the situations that might happen over the course of the disease (Eicher et al., 2015), such as symptoms derived from the treatment, the treatment itself or the cancer diagnosis (Smith et al., 2016). It is therefore essential to help patients believe in their own ability to face the disease in order to improve—or maintain—their quality of life (Hinz et al., 2019). With regard to optimism, results are in line with studies that relate cancer patients’ optimism with higher quality of life (Anderson et al., 2019; Marton et al., 2020). Optimist patients perceive and see events with a more positive attitude, thus reducing stress and acting as a highly relevant predictor for quality of life in cancer patients (Fischer et al., 2018; Jimenez-Fonseca et al., 2018).

Regarding the types of support and patients’ quality of life, we found the most significant ones to be emotional and instrumental support. Results also show that family, partner and friends are the most important sources of support when it comes to patients’ quality of life. However, health professionals as a source of support relates to higher quality of life. Very often, families become patients’ main sources of support (Yağmur and Duman, 2016), reducing stress and increasing patients’ emotional wellbeing (Spatuzzi et al., 2016). This source of support provides both emotional and physical benefits for patients, which have a positive impact on patients’ quality of life (Neris et al., 2020). Cancer survivors often report the importance of emotional support from their partners for coping with the overall disease process, from the initial diagnosis to the treatment administered and the subsequent consequences (Pfaendler et al., 2015). The same applies to emotional (as well as instrumental and informational) support from family and friends (Doran et al., 2019; Adam and Koranteng, 2020), considered essential for the improvement of patients’ quality of life (Zhang et al., 2020).

Informational support is also relevant due to patients needing information to better cope with the disease (So et al., 2014). The role of health professionals is particularly relevant in this area, as they can also contribute to improve patients’ quality of life. In order for informational and emotional support from health professionals to be effective in the increase of patients’ quality of life, high communication between patients and health professionals must take place (Zhang et al., 2020). In fact, many patients report that most of the informational support they receive comes from health professionals (Adam and Koranteng, 2020). Patients consider such support essential to improve their knowledge on the disease and learn how to cope with it more adaptively.

The multidimensional approach of the present study highlights the importance of understating patients’ needs of support so the specific type of support they need from their networks can be provided.

One of the study’s relevant contributions is the inclusion of health professionals as support sources for patients. The high value given by patients to this source must not be disregarded, which is sometimes compared to the support received from friends and family (Sjölander and Berterö, 2008), thus making it a key source of support in crucial stages of the disease such as the initial diagnosis or during treatment.

### Limitations

Due to the cross-sectional nature of the present study, conclusions about the possible variability in time of the support provided to patients, their optimism, resilience and quality of life could not be extracted. Therefore, we suggest for future lines of research to carry out longitudinal studies to observe and assess fluctuations of psychosocial variables during the overall disease process. This would enable analyses and interventions aimed at increasing cancer patients’ quality of life, social support, optimism and resilience based on the different stages of the disease. Based on the study’s results, we suggest creating theoretical models to predict cancer patients’ quality of life considering all the variables analysed.

### Clinical Implications

Considering the importance of improving patients’ quality of life (Sommer et al., 2018), it is key to analyse the psychosocial dimension of support and carry out intervention strategies that focus in improving patients’ quality of life (Marotta et al., 2020). Intervention strategies would need to aim at involving cancer patients’ relatives and friends in the disease process, considering patients’ needs for support. Likewise, interventions to promote support groups are also essential, since they have been observed to be a powerful tool for cancer patients. Such strategies must also aim at improving patients’ resilience and optimism so they can better cope with the disease, thus contributing to increase their quality of life.

## Data Availability Statement

The raw data supporting the conclusions of this article will be made available by the authors, without undue reservation.

## Ethics Statement

The studies involving human participants were reviewed and approved by The Ethical Committee of the University of Málaga, as well as the Hospital Costa del Sol’s. The patients/participants provided their written informed consent to participate in this study.

## Author Contributions

IR-R and IH-M: conceptualization, methodology, and formal analysis. IR-R, IH-M, AM-G and MM-M: investigation, writing-review and editing, and supervision. IR-R: writing-original draft preparation. IH-M: funding acquisition. All authors have read and agreed to the published version of the manuscript.

## Funding

This work was funded by PAIDI research group HUM-590, Junta de Andalucía.

## Conflict of Interest

The authors declare that the research was conducted in the absence of any commercial or financial relationships that could be construed as a potential conflict of interest.

## Publisher’s Note

All claims expressed in this article are solely those of the authors and do not necessarily represent those of their affiliated organizations, or those of the publisher, the editors and the reviewers. Any product that may be evaluated in this article, or claim that may be made by its manufacturer, is not guaranteed or endorsed by the publisher.
